# Ocular symptoms in COVID-19 patients with a history of hospitalization in the first pandemic wave in Styria, Austria

**DOI:** 10.3389/fopht.2025.1540904

**Published:** 2025-02-27

**Authors:** Andreas Guttmann, Astrid Heidinger, Nora Woltsche, Marianne Brodmann, Katharina Kurzmann-Gütl, Viktoria Nemecz, Matthias Kaindl, Herbert Wurzer, Gerold Schwantzer, Jutta Horwath-Winter

**Affiliations:** ^1^ Department of Ophthalmology, Medical University Graz, Graz, Austria; ^2^ Division of Angiology, Medical University Graz, Graz, Austria; ^3^ Department of Internal Medicine, LKH Graz II, Graz, Austria; ^4^ Institute for Medical Informatics, Statistics and Documentation, Medical University Graz, Graz, Austria

**Keywords:** COVID-19, ocular symptoms, prevalence, conjunctivitis, SARS-CoV-2

## Abstract

**Purpose:**

Our study aimed to investigate the prevalence and timing of ocular surface manifestations in hospitalized COVID-19 patients, providing insights into the occurrence of eye involvement before, during, or after the illness. This study contributes to understanding the extent of ocular involvement in COVID-19, which has been suggested to occur due to potential viral entry through the eyes.

**Methods:**

451 confirmed COVID-19 patients had a history of hospitalization in Styria, Austria. The study included 176 patients aged 18-95 years who tested positive for SARS-CoV-2 in nasopharyngeal swabs by RT-PCR and received treatment at two hospitals. Telephone interviews were conducted after recovery, focusing on ocular symptoms and medical history (openMEDOCS).

**Results:**

Seventeen percent (n=30) reported new-onset ocular symptoms in the context of COVID-19. Patients with ocular symptoms were younger (p<0.001). Sore throat (p=0.013) and high fever (p=0.038) were significantly more prevalent in patients with new-onset ocular symptoms. Persistent ocular symptoms beyond the duration of hospitalization affected more than half (56.7%) of the participants with new-onset ocular symptoms. However, there were no differences in blood parameters, lung imaging, or comorbidities between groups with and without ocular symptoms.

**Conclusions:**

In hospitalized COVID-19 patients, ocular symptoms occur with a significant prevalence of 17%. Younger age (p<0.001) and the presence of sore throat (p=0.013) are associated with an increased risk of developing new-onset ocular symptoms in the context of COVID-19.

## Introduction

On January 7th, 2020 a novel type of coronavirus was isolated and confirmed as a pathogen by the Chinese Centre for Disease Control and Prevention and named 2019-nCoV by the World Health Organization (WHO) (1). However, a month later the WHO declared a new name for the pandemic disease: Coronavirus Disease (COVID-19) (2). The Coronavirus Study Group of the International Committee on Taxonomy of Viruses named it Severe Acute Respiratory Syndrome-related Coronavirus 2, or SARS‐CoV‐2 (3).

SARS-CoV shares an 82% genetic similarity with SARS-CoV-2; however, ocular involvement has not been observed in SARS-CoV cases according to current scientific findings (4–6). Similarly, SARS-CoV-2 demonstrates significant neuroinvasive and neurotropic activity, as seen in its association with anosmia, which is hypothesized to arise from its impact on the olfactory neuroepithelium and immune system interactions. These mechanisms underscore its potential to affect sensory systems (7). Polymerase chain reaction (PCR) analysis in tear fluid of SARS-CoV infected patients showed that the virus antigen was actually present there despite the absence of ocular symptoms (OS) (8).

The highly infectious SARS-Cov-2 is mainly transmitted via aerosols (9). Detection of SARS-CoV-2 ribonucleic acid (RNA) in tears and conjunctiva should strengthen ocular transmission, but this seems unusual with a SARS-CoV-2 detection rate of only 2.7% in ocular samples (10).

Various studies have shown that COVID-19 can affect the eyes by causing conjunctivitis or conjunctivitis like symptoms. Ocular surface involvement ranges from 0.8 to 31.6% of cases (11–13). When considering the effects of COVID-19 on the whole eye, ocular manifestations are reported in up to 32% (14). In addition, a few cases were described in which conjunctivitis was observed as the first or main symptom (13, 15, 16).

The aim of our study was to investigate the prevalence of ocular symptoms in COVID-19 patients with a history of hospitalization. Therefore, we conducted a telephone interview with a questionnaire from patients who had already recovered from COVID-19 in order to investigate the occurrence of ocular involvement and if whether it occurred before, during or after the illness. Furthermore, we wanted to evaluate whether there is a connection between the course of the disease and the OS.

## Methods

The study was conducted on the basis of the criteria of the Declaration of Helsinki and approved by the Ethics Committee of the Medical University of Graz (protocol number: EK 32-535 ex 19/20). Verbal informed consent was obtained from the participants at the beginning of the telephone interview.

During the first wave of the COVID-19 pandemic, almost all hospitalized patients in the province of Styria, Austria were treated in two hospitals in their provincial capital Graz, the LKH University Hospital Graz and in the LKH Graz II, Location West. From 7/3/2020 to 10/8/2020, 451 reverse transcriptase-polymerase chain reaction (RT-PCR) confirmed COVID-19 patients were treated on ward or in an intensive care unit at these two hospitals. Therefore, all patients who (1) tested pharyngeal swab positive for the SARS-CoV-2 RNA by RT-PCR and (2) were between 18 and 95 years of age and (3) were treated for COVID-19 at either hospital were included in our study. Excluded were mentally disabled and persons under custodianship, but also those for whom no current contact details were stored. More than one-third (n=104 (37.8%)) were unable to participate in the study because they were deceased at the time of the survey.

### Data collection

The study team obtained the names, dates of birth, and dates of the first positive RT-PCR test results from the treating departments. Additional demographic and medically relevant information, including allergies, past medical history, medication use, admission findings, duration of inpatient stay, radiological and laboratory findings, and other medical interventions, were obtained through the hospital information system openMEDOCS (KAGes, Graz, Austria).

### Questionnaire

To enhance the quality of questionnaire responses, the administration of the questionnaire was conducted through telephone interviews. All patients for whom no exclusion criteria were already present during the data collection process were interviewed exclusively by two residents in ophthalmology. The interviews were conducted between 13^th^ of August 2020 and 19^th^ of December 2020. The participants were assigned a pseudonymization code and this was noted on the questionnaire. The questionnaire was divided into a general and a specific part. In the general part, participants were asked about past medical history, allergies, and duration of COVID-19. In addition, an ophthalmologic history was collected, whether eye diseases exist, eye surgeries were performed, and ophthalmic medication was applied or a refractive correction by glasses or contact lenses was necessary. Furthermore, the participants were asked about their digital screen time, whether it was longer than 5 hours per day.

The specific part was based on the assessment of the following OS: Redness, swelling of the conjunctiva; swelling of the eyelid, redness of the eyelid; itching, burning, foreign body sensation; sensitivity to light; epiphora; stuck eyes; conjunctival secretion; blurred vision and periorbital pain. If symptoms had occurred, participants were asked which eye was affected, the time of onset, and the duration of symptoms in days. We addressed whether these OS occurred and, if so, whether they appeared before or during COVID-19 infection related illness/hospitalization, at the time of discharge or after the hospitalization. Participants rated the intensity of their symptoms on a scale of 0-100, with “0” indicating no symptoms and “100” indicating maximal discomfort. This symptom assessment is based on the Visual Analog Scale (VAS).

For the statistical analysis, the collected data from the hospital information system and the responses from the questionnaire were merged and consolidated.

### Statistical analysis

Categorical data is given as frequency with percentage, continuous data as mean together with standard deviation (SD) or as median together with range (minimum – maximum). Group differences in categorical data were assessed with Chi-squared test, continuous parameters were tested with Mann-Whitney-U test. In both cases we calculated exact test statistics. P-values less than 0.05 were considered statistically significant. Statistical analysis was performed with IBM SPSS Statistics Version 28 (Release 28.0.1.1 2021. Armonk (NY), USA: International Business Machines Corporation).

## Results

A total of 176 (39.0%; male=95 (54%)/female=81(46%)) out of the 451 PCR positive tested patients agreed to answer the questions. 30 patients (17%; m=13 (43.3%)/f=17 (56.7%)) described having suffered from new-onset OS in the context of COVID-19 or still suffering until the time of evaluation.

The mean age of study participants was 64.6 ± 15.37 years (mean [SD]), with a range of 20 to 93 years.

Statistically, there was a significant difference in age (p<0.001) between the group with new-onset OS and the group without. Patients with new onset OS were younger (55.7 ± 15.3) than patients without OS in the context of COVID-19 (66.4 ± 14.8) (**Table 1**).

**Table 1:** Characteristics of patients with and without new-onset ocular symptoms.

	with new-onset OS (n, Mean ± SD or %)	without new-onset OS (n, Mean ± SD or %)	p-value
Age (years)	30, 55.67 ± 15.27	146, 66.38 ± 14.80	<0.001
Sore Throat			
Yes	3 (11.1%)	1 (0.7%)	0.013
No	24 (88.9%)	140 (99.3%)	–
Cephalalgia			
Yes	4 (14.8%)	7 (5.0%)	0.079
No	23 (85.2%)	134 (95.0%)	–
Myalgia or Arthralgia			
Yes	4 (14.8%)	6 (4.3%)	0.056
No	23 (85.2%)	135 (95.7%)	–
General Leading Symptoms upon Hospital Admission			
Cough	13 (48.1%)	80 (56.7%)	0.527
Fatigue	14 (51.9%)	77 (54.6%)	0.835
Dyspnoea	14 (51.9%)	56 (39.7%)	0.289
Fever (≥37.3°C)	9 (36%)	47 (34.9%)	0.636
Gastrointestinal Symptoms	6 (22.2%)	47 (33.3%)	0.273
Digital Screen Time			
>5 hours	9 (30.0%)	32 (21.9%)	0.349
<5 hours	21 (70.0%)	114 (78.1%)	–
Fever >39.0°C			
OS at Admission	2 (15.4%)	4 (2.7%)	0.075
OS at Discharge	2 (22.2%)	4 (2.6%)	0.038
OS after Discharge	2 (14.3%)	4 (2.7%)	0.102
Oxygen Therapy			
OS at Discharge	8 (88.9%)	107 (67.3%)	0.275
OS after Discharge	13 (92.9%)	102 (66.2%)	0.067
Location of Symptoms			
Both Eyes	43 (70.5%)	–	–
Right Eye	5 (8.2%)	–	–
Left Eye	13 (21.3%)	–	–

This table summarizes the characteristics and key symptoms of patients with and without new-onset ocular symptoms (OS), including their prevalence, associations with clinical parameters, and statistical significance.

Of the 176 participants, 31 (17.6%; 4 with and 27 without new-onset OS, p=0.607) required at least temporary transfer to an intensive care unit, whereas the remainder could be cared for as inpatients in one of the two hospitals. Forty-one (24.3%) patients stated that they have allergies with no difference between the group reporting new-onset OS (n=7, 25.9%) and those reporting no OS (n=34, 23.3%).

One hundred fifteen (66.9%) patients (15 in the OS group and 100 in the patients without OS) reported general diseases in cardiovascular and cerebrovascular diseases and in diabetes mellitus. Four (26.7%) of the 15 OS patients with general diseases reported cardiovascular and cerebrovascular diseases compared to 55 (55.0%) of the 100 patients with general symptoms in the group without OS (p=0.053).

Only one (6.7%) patient with general diseases in the OS group reported diabetes mellitus whereas 30 (30.0%) patients without OS did (p=0.066). There are no differences between the groups in the presence of skin diseases or whether systemic medication is taken.

The leading symptom at the time of inpatient admission sore throat was significantly more prevalent in the group with OS (p=0.013). Patients who had new-onset OS were more likely to report cephalalgia (p=0.079) and myalgia or arthralgia (p=0.056) than in the group without new-onset OS. (**Table 1**) The most commonly reported general leading symptoms upon hospital admission among the 176 study participants were cough (n=93 (55.4%)), fatigue (n=91 (54.2%)), dyspnea (n=70 (41.7%)), fever ≥37.3 degrees Celsius (n=56 (35%)), and gastrointestinal symptoms (n=53 (31.5%)). These symptoms are presented separately for participants with and without OS in **Table 1** to allow for a clearer comparison of their prevalence in both groups.

Neither the lung X-rays nor the thoracic computed tomography (CT) scans demonstrated any significant differences between the patient groups with and without new-onset OS. The blood parameters taken at the beginning of the inpatient admission presented in **Table 2** did not indicate any differences between the two groups.

**Table 2:** Differential blood parameters in COVID-19 patients.

	with new-onset ocular symptoms					without new-onset ocular symptoms			p-value
	n	Median	Min	Max	n	Median	Min	Max	
Lymphocytes (in %)	25	30	4	48.8	130	26	10	59	0.239
C-reactive protein (in mg/dl)	25	9.1	0.1	51	130	9.47	0.1	64.12	0.643
Interleukin-6 (in pg/ml)	11	49.1	2.2	3190	34	111	11.5	4980	0.277
Ferritin (in ng/ml)	19	1044.37	72	11424.9	93	1113.88	40.32	15452	0.503
D-dimers (in µg/l)	13	1310	330	64568	71	1393.00	302	54500	0.940

Blood parameters are compared in terms of number, median, minimum, and maximum values between patients with new-onset ocular symptoms and those without. Additionally, the p-values (determined through the Mann-Whitney U test) are reported.

The OS, their frequency and the mean, minimum and maximum intensity of symptoms are characterized in **Table 3**. Blurred vision emerged as the predominant symptom, while peribulbar pain was the most affecting symptom in mean intensity. The majority of symptoms are clearly expressed in both eyes (n=43 (70.5%)) whereas 21.3% (n=13) occur strictly in the left or 8.2% (n=5) in the right eye (**Table 1**).

**Table 3:** Frequency and severity of new-onset ocular symptoms in COVID-19 Patients.

Ocular Symptoms	Number of Patients Reporting Symptoms	Total Nominations of Symptoms (Across All Time Points)	Mean VAS	Min VAS	Max VAS
Redness, Swelling of the conjunctiva	7 (15%)	11 (13%)	30.00	5	80
Swelling of the eyelid/Redness of the eyelid	0 (0%)	0 (0%)			
Itching, Burning, Foreign body sensation	11 (24%)	17 (19%)	43.52	10	70
Sensitivity to light	6 (13%)	16 (18%)	50.00	10	80
Epiphora	7 (15%)	15 (17%)	38.57	10	70
Stuck eyes	1 (2%)	2 (2%)	30.00	30	30
Conjunctival secretion	0 (0%)	0 (0%)			
Blurred vision	11 (24%)	24 (27%)	42.88	10	90
Peribulbar pain	3 (7%)	3 (3%)	56.67	50	60

Mean, minimum and maximum VAS-scores for specific ocular symptoms reported by how many patients across all rated time points. Mean VAS values are obtained by averaging each patient’s mean of 1 up to 4 rated time points - before, during, at and after in-patient stay (mean of patient’s means).

**Figure 1:**
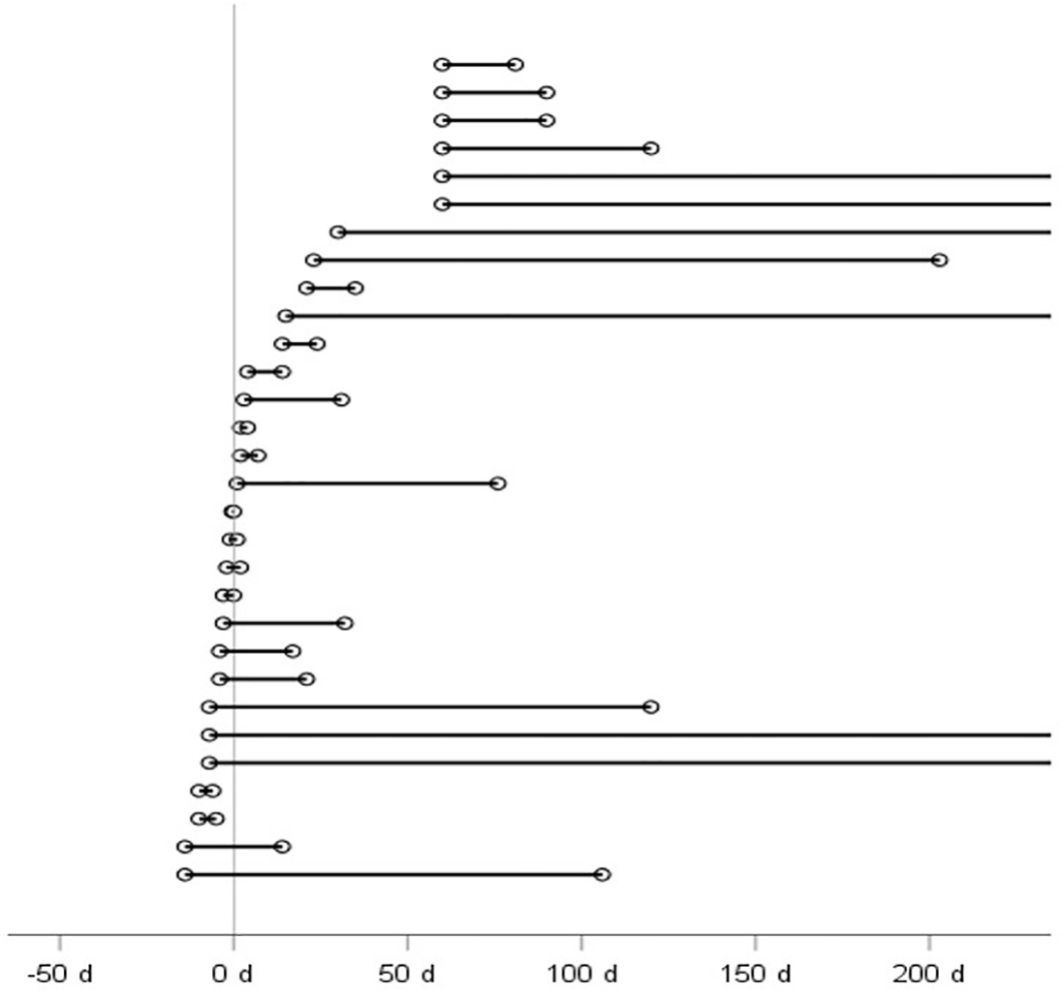
Patient´s duration of ocular symptoms in days (d) in relation to COVID-19 onset (0 d). Each line represents a patient with new-onset ocular symptoms. Because patients who experienced new-onset OS after their hospital stay could not recall the exact timing of onset at the time of the telephone interview, the 60th day post-discharge was chosen as the time of onset.

The duration of OS of each patient is shown in **Figure 1**. 14 (46.7%) patients reported OS as the initial symptom. On average, 6.2 ± 4.5 days with a maximum of 14 days prior to first COVID symptom. The participants in our study reported a duration of OS of 28 days (min=0.5 d). OS that emerged after discharge from the inpatient stay lasted for a longer duration of 45 days (min=21 d, max=305 d) compared to those that occurred prior to the onset of COVID-19, which lasted for 23 days (min=0.5 d, max=372 d), or those that occurred during the inpatient stay, which lasted for 21 days (min=2 d, max=350 d). Persistent OS beyond the duration of hospitalization affected more than half (n=17 (56.7%)) of the participants with new-onset OS. Treatment with preservative-free lubricants during the inpatient stay was necessary in three patients (10%).

Ten patients (5.7%) reported regular contact lens use. In two of them, both using one-day contact lenses, new-onset OS occurred in the context of COVID-19. None of the two patients rated their contact lens compatibility as “good” whereas each of the 8 patients without new-onset OS did (p=0.022). About one-fourth (n=41 (23.3%)) of all participants reported more than 5 hours of digital screen time per day. However, it has no influence on the occurrence of symptoms between the two groups (p=0.349). Four participants responded suffering from diagnosed dry eye disease (DED) but did not report any new onset of eye symptoms or increase in their symptoms.

Moreover, the need for oxygen therapy in 18 (69.2%) patients who had new-onset OS, had no significant effect on the occurrence of OS. However, patients with need for oxygen therapy are more likely to get or maintain OS after discharge (p=0.067).(**Table 1**) Special intensive care positioning (e.g.: prone position) was only necessary in one patient who reported OS.

Patients with fever >39.0 degrees Celsius upon hospital admission were more likely to develop OS during inpatient stay (p=0.075). The presence of new-onset OS at discharge was significantly influenced (p=0.038) by high fever as a leading symptom upon hospital admission. (**Table 1**) Thus, in this group reporting OS at discharge, 22.2% (n=2) presented with a temperature >39.0 degrees Celsius, whereas in the group without OS only 2.6% (n=4) were in this temperature range.

## Discussion

In our study we could show that 17% (n=30/176) COVID-19 patients with a history of hospitalization had suffered from new-onset OS or were still suffering until the time of evaluation. To the best of our knowledge, this is the largest study focusing on the prevalence of ocular symptoms in hospitalized patients with COVID 19 with results displaying the middle range of the previous results published in the literature (11, 13).

Although participants from only two hospitals were interviewed, they provide a good reflection of the population, with a large number of participants (n=176), a wide age range (20 to 93 years), and a nearly even gender distribution (f=46.0% vs. m=54.0%).

As defined in the TFOS DEWS II, “Dry eye is a multifactorial disease of the tears and ocular surface that results in symptoms of discomfort, visual disturbance, and tear film instability with potential damage to the ocular surface. It is accompanied by increased osmolarity of the tear film and inflammation of the ocular surface.” (17) Due to this multifactoriality, it can of course not be completely ruled out that the symptoms could have been triggered by another cause than COVID-19. The positioning of patients, especially the prone position, leads to an increased risk of keratopathy and thus to the occurrence of DED (18, 19). We could not detect any indication that prone position was the cause for the occurrence of new OS, as only one patient out of seven who had to be positioned in this way during the inpatient stay developed such symptoms. Unlike other studies that suggest a link between digital screen time and the development of DED (20, 21), our research shows that digital screen time has no impact on the development of new-onset OS before, during or after COVID-19 disease. (**Table 1**) The need for oxygen therapy appears to facilitate the development of new OS following hospital discharge. However, in our study, it appears that this is not the primary reason for such symptoms. Acar et al. demonstrated in their study that positive airway pressure ventilation has a substantial long-term impact on the ocular surface, over a period of 18 months (22). In our study, the duration and type of oxygen ventilation could not be determined retrospectively, as these data were not obtainable.

Improved methods have now also been used to detect SARS-CoV-2 in the conjunctival swab in over 50% of hospitalized patients (23–25). The observed significant correlation between new-onset OS and sore throat in our study group supports the plausibility of a dynamic infection route involving the eyes (26, 27). The fact that 46.7% of our patients with new-onset OS presented them as the initial symptom up to 14 days prior to a positive RT-PCR test for SARS-CoV-2 also supports the possibility of ocular transmission of the virus.

The presence of high fever (>39.0 degrees Celsius) may increase the likelihood of OS in COVID-19 patients, during the inpatient stay (p=0.075) and significantly on admission to the hospital (p= 0.038). Further research is needed to better understand the underlying mechanisms of this association and whether early intervention for fever may help prevent OS in COVID-19 patients. Unlike the study by Chen et al., our study did not find any trend between radiologic findings on lung X-ray and CT and the occurrence of OS (28).

As we now know, COVID-19 on the outer eye may additionally present with other clinical pictures, such as otherwise unexplained new-onset stromal corneal opacities which resolved on steroid therapy, suggesting an underlying inflammatory or immune-mediated mechanism. Given their response to steroids and similarities to epidemic keratoconjunctivitis, Pareja-Ríos et al. suggest that these findings might be immunologically related or represent incidental but unusual associations with other viral infections (29). Notably, the impact of the disease extends beyond the ocular surface, with documented effects in various regions of the entire eye. A comprehensive compilation of these effects has been presented in a review by Sen et al. (30).

Complaints of DED seem to appear more frequently, likely attributable to increased face mask use, which has been shown to exacerbate ocular surface symptoms through altered airflow dynamics (31–33). White (34) described this etiology of dry eye as mask-associated dry eye, or MADE. In addition, face masks can also have an influence on the results of examinations, such as the Visual Field Scores (35). But MADE had no influence on the occurrence of new OS before and during hospitalization in our patients, as the mask requirement imposed by the federal government in Austria came into effect late during the first wave of the pandemic. However, it is possible that the mask may have triggered the onset of symptoms after the patient’s discharge from the hospital.

## Limitations

Our study has several limitations. Given the novelty of the disease, which was poorly understood at the time, the strict preventive measures in Austria, and the need to ensure the safety of the study team, a telephone questionnaire as study design was the only feasible option for a safe data collection. Nonetheless, this approach allowed us to achieve a response rate of 39%.

The interviews conducted several months after inpatient discharge resulted in ambiguous statements from some patients. Specifically, those who developed new-onset OS after discharge were unable to provide a precise time of onset.

At the onset of the pandemic, due to limited knowledge and the urgent need to conserve resources, there were disparities in data collection practices across the two hospitals. As a result, progression or certain relevant parameters, such as interleukin-6, were not included in all patients as they were only measured in selected cases.

## Conclusion

In conclusion, our study provides important insights into the prevalence and characteristics of new-onset OS in hospitalized COVID-19 patients. The results indicate that 17% of patients suffer from ocular surface symptoms, which is consistent with previous studies in the literature (11, 13). Furthermore, the study highlights the possibility of ocular transmission of SARS-CoV-2 and the potential association between high fever and OS. While our study has some limitations, including the study design and disparities in data collection practices, it represents the largest study focusing on ocular symptoms in hospitalized COVID-19 patients to date. These findings underscore the importance of recognizing OS in COVID-19 patients, advocating for routine screening and prompt management to enhance patient outcomes and comfort. There is a need for further research to better understand the mechanisms underlying the relationship between COVID-19 and ocular symptoms.

## Data Availability

The raw data supporting the conclusions of this article will be made available by the authors upon reasonable request.
